# Supplementary material for: In Vitro Development of Local Antiviral Formulations with Potent Virucidal Activity Against SARS-CoV-2 and Influenza Viruses
Source: Pharmaceutics. 2025 Mar 8;17(3):349. doi: 10.3390/pharmaceutics17030349 (PMC11945346; doi:10.3390/pharmaceutics17030349)
Supplement: Supplementary file 1 [file pharmaceutics-17-00349-s001.zip › pharmaceutics-3486651-supplementary.pdf]

pharmaceutics

MDPI

Supplementary Materials

<table>
<tr><th>Surfactant</th><th>Time</th><th>Ratio of DLM to surfactant</th></tr>
<tr><td rowspan="2">Tween 20</td><td>1 Day after preparation</td><td>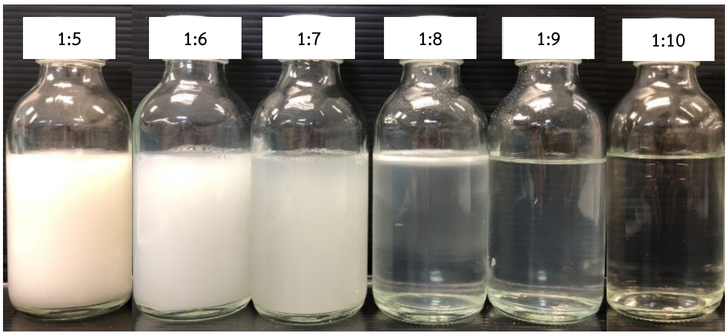
</td></tr>
<tr><td>After a temperature cycling test (6 cycles)</td><td>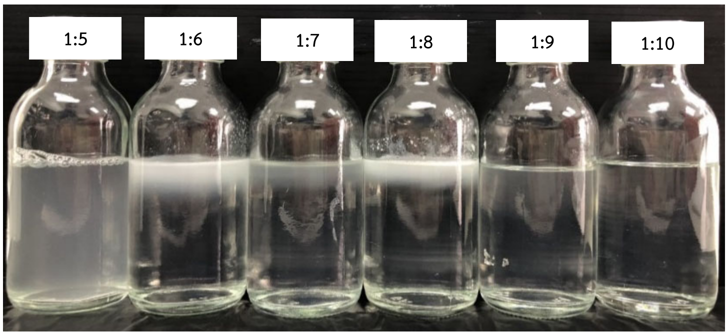
</td></tr>
<tr><td rowspan="2">Tween 60</td><td>1 Day after preparation</td><td>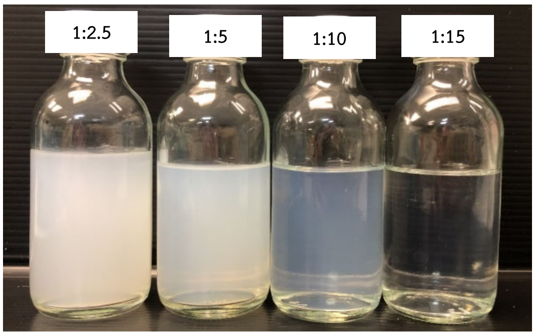
</td></tr>
<tr><td>After a temperature cycling test (6 cycles)</td><td>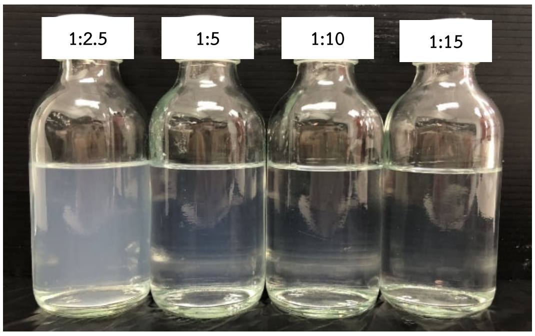
</td></tr>
</table>

Supplementary Figure S1. Physical characteristics (turbid or clear) of formulations containing DLM and surfactant at different ratios, measured 1 day after preparation and after a temperature cycling test (6 cycles).

<table>
<tr><th>Surfactant</th><th>Time</th><th>Ratio of DLM to surfactant</th></tr>
<tr><td rowspan="2">Tween 80</td><td>1 Day after preparation</td><td>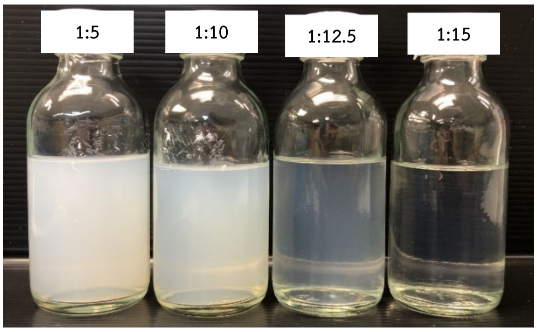
</td></tr>
<tr><td>After a temperature cycling test (6 cycles)</td><td>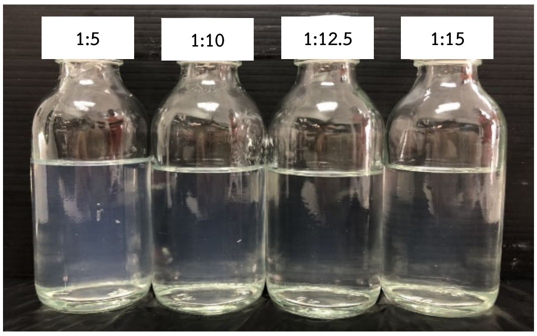
</td></tr>
<tr><td rowspan="2">Cremophor RH40</td><td>1 Day after preparation</td><td>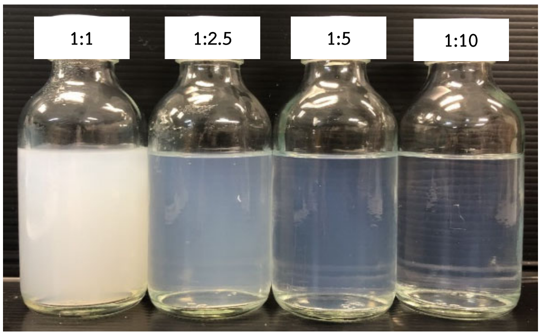
</td></tr>
<tr><td>After a temperature cycling test (6 cycles)</td><td>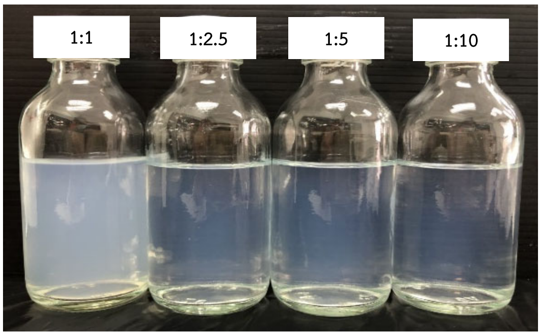
</td></tr>
</table>

Supplementary Figure S1. *Cont.* Physical characteristics (turbid or clear) of formulations containing DLM and surfactant at different ratios, measured 1 day after preparation and after a temperature cycling test (6 cycles).

Surfactant	Time	Ratio of DLM to surfactant
Cremophor RH60	1 Day after preparation	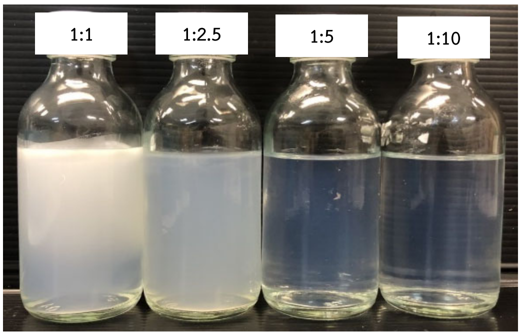
	After a temperature cycling test (6 cycles)	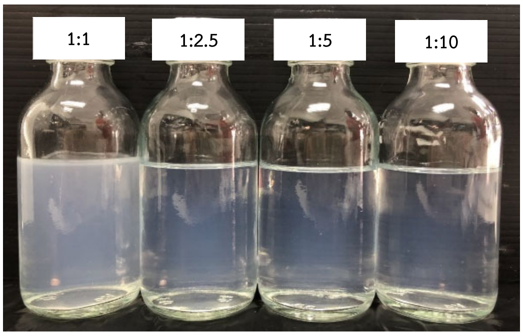
Coco glucoside	1 Day after preparation	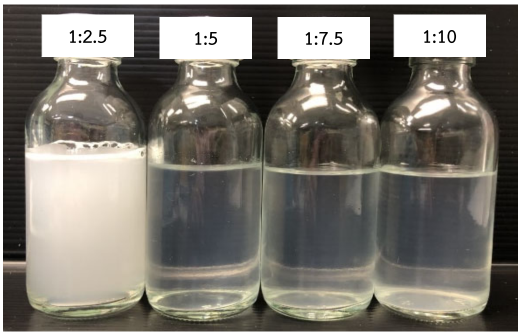
	After a temperature cycling test (6 cycles)	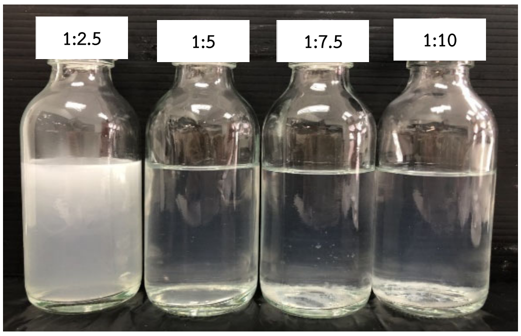

Supplementary Figure S1. *Cont.* Physical characteristics (turbid or clear) of formulations containing DLM and surfactant at different ratios, measured 1 day after preparation and after a temperature cycling test (6 cycles).

Surfactant	Time	Ratio of DLM to surfactant
Decyl glucoside	1 Day after preparation	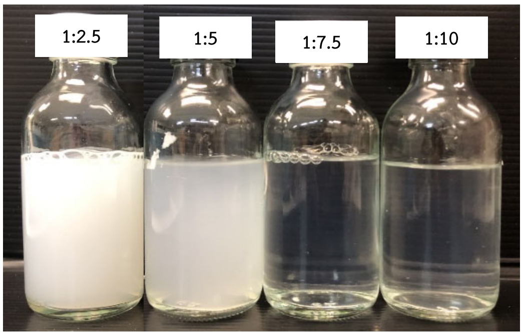
	After a temperature cycling test (6 cycles)	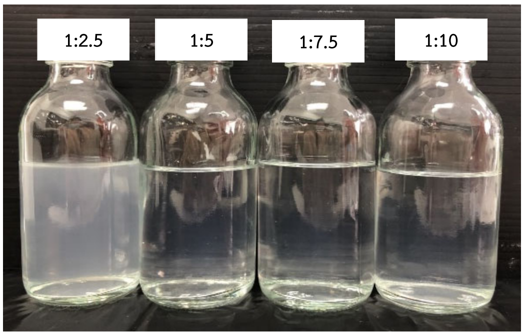

Supplementary Figure S1. *Cont.* Physical characteristics (turbid or clear) of formulations containing DLM and surfactant at different ratios, measured 1 day after preparation and after a temperature cycling test (6 cycles).

Supplementary Table S1. Cytotoxicity and neutralization validation control data for oral formulation D in MDCK cells.

Dilution	Cytotoxicity set 1				Cytotoxicity set 2				Cytotoxicity set 3				Cytotoxicity set 4			
10^{-1}	0	0	0	0	0	0	0	0	0	0	0	0	0	0	0	0
10^{-2}	0	0	0	0	0	0	0	0	0	0	0	0	0	0	0	0
10^{-3}	0	0	0	0	0	0	0	0	0	0	0	0	0	0	0	0
10^{-4}	0	0	0	0	0	0	0	0	0	0	0	0	0	0	0	0
10^{-5}	0	0	0	0	0	0	0	0	0	0	0	0	0	0	0	0
10^{-6}	0	0	0	0	0	0	0	0	0	0	0	0	0	0	0	0
10^{-7}	0	0	0	0	0	0	0	0	0	0	0	0	0	0	0	0
10^{-8}	0	0	0	0	0	0	0	0	0	0	0	0	0	0	0	0

Note: Key: "+" = Virus recovery; "0" = No virus recovery and/or no cytotoxicity observed; "T" = Toxicity observed.

Supplementary Table S2. Cytotoxicity and neutralization validation control data for nasal formulation E in MDCK cells.

Dilution	Cytotoxicity set 1				Cytotoxicity set 2				Cytotoxicity set 3				Cytotoxicity set 4			
10^{-1}	0	0	0	0	0	0	0	0	0	0	0	0	0	0	0	0
10^{-2}	0	0	0	0	0	0	0	0	0	0	0	0	0	0	0	0
10^{-3}	0	0	0	0	0	0	0	0	0	0	0	0	0	0	0	0
10^{-4}	0	0	0	0	0	0	0	0	0	0	0	0	0	0	0	0
10^{-5}	0	0	0	0	0	0	0	0	0	0	0	0	0	0	0	0
10^{-6}	0	0	0	0	0	0	0	0	0	0	0	0	0	0	0	0
10^{-7}	0	0	0	0	0	0	0	0	0	0	0	0	0	0	0	0
10^{-8}	0	0	0	0	0	0	0	0	0	0	0	0	0	0	0	0

Note: Key: "+" = Virus recovery; "0" = No virus recovery and/or no cytotoxicity observed; "T" = Toxicity observed.

Supplementary Table S3. Test results for oral formulation D and nasal formulation E at a dilution of 1:32 against FluA(H1N1pdm) (A/Thailand/104/2009).

Test Product	Concentration	Virus titer ($TCID_{50}$)	Log_{10} Reduction	% Reduction
Oral formulation D	1:32	$3.16x10^2$	>4.000	>99.99%
Nasal formulation E	1:32	$1.93x10^2$	>4.000	>99.99%
Tested Virus	FluA(H1N1pdm) (A/Thailand/104/2009)			
Virus Control	$1x10^7$ $TCID_{50}$			
Virus recovery	$4.25x10^6$ $TCID_{50}$			
Cytotoxicity Control	No cytotoxicity observed			
Sterility Control	No contamination observed			

Supplementary Table S4. Virus recovery data for FluA(H1N1pdm) (A/Thailand/104/2009).

Dilution	Virus control set 1				Virus control set 2				Virus control set 3				Virus control set 4			
10^{-1}	+	+	+	+	+	+	+	+	+	+	+	+	+	+	+	+
10^{-2}	+	+	+	+	+	+	+	+	+	+	+	+	+	+	+	+
10^{-3}	+	+	+	+	+	+	+	+	+	+	+	+	+	+	+	+
10^{-4}	+	+	+	+	+	+	+	+	+	+	+	+	+	+	+	+
10^{-5}	+	+	+	+	+	+	+	+	+	+	+	+	+	+	+	+
10^{-6}	+	0	0	0	0	0	0	0	+	0	0	0	+	0	0	0
10^{-7}	0	0	0	0	0	0	0	0	0	0	0	0	0	0	0	0
10^{-8}	0	0	0	0	0	0	0	0	0	0	0	0	0	0	0	0
Log	$5.62x10^{6}$				$3.16x10^{6}$				$5.62x10^{6}$				$5.62x10^{6}$			
Average	$4.25x10^{6}$															

Supplementary Table S5. Virucidal activity of oral formulation D at a dilution of 1:32 against FluA(H1N1pdm) (A/Thailand/104/2009).

Dilution	Virus-test suspension set 1				Virus-test suspension set 2				Virus-test suspension set 3				Virus-test suspension set 4			
10^{-1}	+	+	+	+	+	+	+	+	+	+	+	0	+	+	+	+
10^{-2}	0	0	0	0	0	0	0	0	0	0	0	0	0	0	0	0
10^{-3}	0	0	0	0	0	0	0	0	0	0	0	0	0	0	0	0
10^{-4}	0	0	0	0	0	0	0	0	0	0	0	0	0	0	0	0
10^{-5}	0	0	0	0	0	0	0	0	0	0	0	0	0	0	0	0
10^{-6}	0	0	0	0	0	0	0	0	0	0	0	0	0	0	0	0
10^{-7}	0	0	0	0	0	0	0	0	0	0	0	0	0	0	0	0
10^{-8}	0	0	0	0	0	0	0	0	0	0	0	0	0	0	0	0
$TCID_{50}$	$3.16x10^{2}$				$3.16x10^{2}$				$3.16x10^{2}$				$3.16x10^{2}$			
Average	$3.16x10^{2}$															

Note: Key: "+" = Virus recovery; "0" = No virus recovery and/or no cytotoxicity observed; "T" = Toxicity observed.

Supplementary Table S6. Virucidal activity of nasal formulation E at a dilution of 1:32 against FluA(H1N1pdm) (A/Thailand/104/2009).

Dilution	Virus-test suspension set 1				Virus-test suspension set 2				Virus-test suspension set 3				Virus-test suspension set 4			
10^{-1}	+	+	0	0	+	+	+	0	+	+	+	0	+	+	+	+
10^{-2}	0	0	0	0	0	0	0	0	0	0	0	0	0	0	0	0
10^{-3}	0	0	0	0	0	0	0	0	0	0	0	0	0	0	0	0
10^{-4}	0	0	0	0	0	0	0	0	0	0	0	0	0	0	0	0
10^{-5}	0	0	0	0	0	0	0	0	0	0	0	0	0	0	0	0
10^{-6}	0	0	0	0	0	0	0	0	0	0	0	0	0	0	0	0
10^{-7}	0	0	0	0	0	0	0	0	0	0	0	0	0	0	0	0
10^{-8}	0	0	0	0	0	0	0	0	0	0	0	0	0	0	0	0
$TCID_{50}$	1.00×10^2				1.78×10^2				1.78×10^2				3.16×10^2			
**Average**	1.93×10^2															

Note: Key: "+" = Virus recovery; "0" = No virus recovery and/or no cytotoxicity observed; "T" = Toxicity observed.

Supplementary Table S7. Test results for oral formulation D and nasal formulation E at a dilution of 1:32 against FluA(H3N2) (ATCC VR-1881™).

Test Product	Concentration	Virus titer ($TCID_{50}$)	Log_{10} reduction	% Reduction
Oral formulation D	1:32	3.775×10^2	>4.000	>99.99%
Nasal formulation E	1:32	5.005×10^2	>4.000	>99.99%
Tested Virus	FluA(H3N2) (ATCC VR-1881™)			
Virus Control	1.25×10^7 $TCID_{50}$			
Virus recovery	5.005×10^6 $TCID_{50}$			
Cytotoxicity Control	No cytotoxicity observed			
Sterility Control	No contamination observed			

Supplementary Table S8. Virus recovery data for FluA(H3N2) (ATCC VR-1881™).

Dilution	Virus control set 1				Virus control set 2				Virus control set 3				Virus control set 4			
10^{-1}	+	+	+	+	+	+	+	+	+	+	+	+	+	+	+	+
10^{-2}	+	+	+	+	+	+	+	+	+	+	+	+	+	+	+	+
10^{-3}	+	+	+	+	+	+	+	+	+	+	+	+	+	+	+	+
10^{-4}	+	+	+	+	+	+	+	+	+	+	+	+	+	+	+	+
10^{-5}	+	+	+	+	+	+	+	+	+	+	+	+	+	+	+	+
10^{-6}	+	0	0	0	0	0	0	0	+	0	0	0	+	0	0	0
10^{-7}	0	0	0	0	0	0	0	0	0	0	0	0	0	0	0	0
10^{-8}	0	0	0	0	0	0	0	0	0	0	0	0	0	0	0	0
Log	5.62×10^6				3.16×10^6				5.62×10^6				5.62×10^6			
Average	5.005×10^6															

Supplementary Table S9. Virucidal activity of oral formulation D at a dilution of 1:32 against FluA(H3N2) (ATCC VR-1881™).

Dilution	Virus-test suspension set 1				Virus-test suspension set 2				Virus-test suspension set 3				Virus-test suspension set 4			
10^{-1}	+	+	+	+	+	+	+	+	+	+	+	0	+	+	+	+
10^{-2}	+	0	0	0	0	0	0	0	0	0	0	0	+	0	0	0
10^{-3}	0	0	0	0	0	0	0	0	0	0	0	0	0	0	0	0
10^{-4}	0	0	0	0	0	0	0	0	0	0	0	0	0	0	0	0
10^{-5}	0	0	0	0	0	0	0	0	0	0	0	0	0	0	0	0
10^{-6}	0	0	0	0	0	0	0	0	0	0	0	0	0	0	0	0
10^{-7}	0	0	0	0	0	0	0	0	0	0	0	0	0	0	0	0
10^{-8}	0	0	0	0	0	0	0	0	0	0	0	0	0	0	0	0
$TCID_{50}$	$5.62x10^2$				$3.16x10^2$				$3.16x10^2$				$3.16x10^2$			
Average	$3.775x10^2$															

Note: Key: "+" = Virus recovery; "0" = No virus recovery and/or no cytotoxicity observed; "T" = Toxicity observed.

Supplementary Table S10. Virucidal activity of nasal formulation E at a dilution of 1:32 against FluA(H3N2) (ATCC VR-1881™).

Dilution	Virus-test suspension set 1				Virus-test suspension set 2				Virus-test suspension set 3				Virus-test suspension set 4			
10^{-1}	+	+	+	+	+	+	+	+	+	+	+	0	+	+	+	+
10^{-2}	+	0	0	0	+	0	0	0	0	0	0	0	+	0	0	0
10^{-3}	0	0	0	0	0	0	0	0	0	0	0	0	0	0	0	0
10^{-4}	0	0	0	0	0	0	0	0	0	0	0	0	0	0	0	0
10^{-5}	0	0	0	0	0	0	0	0	0	0	0	0	0	0	0	0
10^{-6}	0	0	0	0	0	0	0	0	0	0	0	0	0	0	0	0
10^{-7}	0	0	0	0	0	0	0	0	0	0	0	0	0	0	0	0
10^{-8}	0	0	0	0	0	0	0	0	0	0	0	0	0	0	0	0
$TCID_{50}$	$5.62x10^2$				$5.62x10^2$				$3.16x10^2$				$5.62x10^2$			
Average	$5.005x10^2$															

Note: Key: "+" = Virus recovery; "0" = No virus recovery and/or no cytotoxicity observed; "T" = Toxicity observed.

Supplementary Table S11. Test results for oral formulation D and nasal formulation E at a dilution of 1:32 against FluB (ATCC VR-1735™).

Test product	Concentration	Virus titer ($TCID_{50}$)	Log_{10} reduction	% Reduction
Oral formulation D	1:32	5.485×10^2	>4.000	>99.99%
Nasal formulation E	1:32	4.390×10^2	>4.000	>99.99%
Tested Virus	FluB (ATCC VR-1735™)			
Virus Control	1.2×10^7 $TCID_{50}$			
Virus recovery	6.10×10^6 $TCID_{50}$			
Cytotoxicity Control	No cytotoxicity observed			
Sterility Control	No contamination observed			

Supplementary Table S12. Virus recovery data for FluB (ATCC VR-1735™).

Dilution	Virus control set 1				Virus control set 2				Virus control set 3				Virus control set 4			
10^{-1}	+	+	+	+	+	+	+	+	+	+	+	+	+	+	+	+
10^{-2}	+	+	+	+	+	+	+	+	+	+	+	+	+	+	+	+
10^{-3}	+	+	+	+	+	+	+	+	+	+	+	+	+	+	+	+
10^{-4}	+	+	+	+	+	+	+	+	+	+	+	+	+	+	+	+
10^{-5}	+	+	+	+	+	+	+	+	+	+	+	+	+	+	+	+
10^{-6}	+	+	0	0	0	0	0	0	+	0	0	0	+	0	0	0
10^{-7}	0	0	0	0	0	0	0	0	0	0	0	0	0	0	0	0
10^{-8}	0	0	0	0	0	0	0	0	0	0	0	0	0	0	0	0
Log	1.00×10^7				3.16×10^6				5.62×10^6				5.62×10^6			
Average	6.10×10^6															

Supplementary Table S13. Virucidal activity of oral formulation D at a dilution of 1:32 against FluB (ATCC VR-1735™).

Dilution	Virus-test suspension set 1				Virus-test suspension set 2				Virus-test suspension set 3				Virus-test suspension set 4			
10^{-1}	+	+	+	+	+	+	+	+	+	+	+	0	+	+	+	+
10^{-2}	+	0	0	0	0	0	0	0	0	0	0	0	+	+	0	0
10^{-3}	0	0	0	0	0	0	0	0	0	0	0	0	0	0	0	0
10^{-4}	0	0	0	0	0	0	0	0	0	0	0	0	0	0	0	0
10^{-5}	0	0	0	0	0	0	0	0	0	0	0	0	0	0	0	0
10^{-6}	0	0	0	0	0	0	0	0	0	0	0	0	0	0	0	0
10^{-7}	0	0	0	0	0	0	0	0	0	0	0	0	0	0	0	0
10^{-8}	0	0	0	0	0	0	0	0	0	0	0	0	0	0	0	0
$TCID_{50}$	5.62×10^2				3.16×10^2				3.16×10^2				1.00×10^3			
Average	5.485×10^2															

Note: Key: "+" = Virus recovery; "0" = No virus recovery and/or no cytotoxicity observed; "T" = Toxicity observed.

Supplementary Table S14. Virucidal activity of nasal formulation E at a dilution of 1:32 against FluB (ATCC VR-1735™).

Dilution	Virus-test suspension set 1				Virus-test suspension set 2				Virus-test suspension set 3				Virus-test suspension set 4			
10^{-1}	+	+	+	+	+	+	+	+	+	+	+	0	+	+	+	+
10^{-2}	+	0	0	0	0	0	0	0	0	0	0	0	+	0	0	0
10^{-3}	0	0	0	0	0	0	0	0	0	0	0	0	0	0	0	0
10^{-4}	0	0	0	0	0	0	0	0	0	0	0	0	0	0	0	0
10^{-5}	0	0	0	0	0	0	0	0	0	0	0	0	0	0	0	0
10^{-6}	0	0	0	0	0	0	0	0	0	0	0	0	0	0	0	0
10^{-7}	0	0	0	0	0	0	0	0	0	0	0	0	0	0	0	0
10^{-8}	0	0	0	0	0	0	0	0	0	0	0	0	0	0	0	0
$TCID_{50}$	5.62×10^2				3.16×10^2				3.16×10^2				5.62×10^2			
**Average**	4.39×10^2															

Note: Key: "+" = Virus recovery; "0" = No virus recovery and/or no cytotoxicity observed; "T" = Toxicity observed.

Supplementary Table S15. Cytotoxicity and neutralization validation control data for oral formulation D in Vero cells.

Dilution	Cytotoxicity set 1				Cytotoxicity set 2				Cytotoxicity set 3				Cytotoxicity set 4			
10^{-1}	0	0	0	0	0	0	0	0	0	0	0	0	0	0	0	0
10^{-2}	0	0	0	0	0	0	0	0	0	0	0	0	0	0	0	0
10^{-3}	0	0	0	0	0	0	0	0	0	0	0	0	0	0	0	0
10^{-4}	0	0	0	0	0	0	0	0	0	0	0	0	0	0	0	0
10^{-5}	0	0	0	0	0	0	0	0	0	0	0	0	0	0	0	0
10^{-6}	0	0	0	0	0	0	0	0	0	0	0	0	0	0	0	0
10^{-7}	0	0	0	0	0	0	0	0	0	0	0	0	0	0	0	0
10^{-8}	0	0	0	0	0	0	0	0	0	0	0	0	0	0	0	0

Note: Key: "+" = Virus recovery; "0" = No virus recovery and/or no cytotoxicity observed; "T" = Toxicity observed.

Supplementary Table S16. Cytotoxicity and neutralization validation control data for nasal formulation E in Vero cells.

Dilution	Cytotoxicity set 1				Cytotoxicity set 2				Cytotoxicity set 3				Cytotoxicity set 4			
10^{-1}	0	0	0	0	0	0	0	0	0	0	0	0	0	0	0	0
10^{-2}	0	0	0	0	0	0	0	0	0	0	0	0	0	0	0	0
10^{-3}	0	0	0	0	0	0	0	0	0	0	0	0	0	0	0	0
10^{-4}	0	0	0	0	0	0	0	0	0	0	0	0	0	0	0	0
10^{-5}	0	0	0	0	0	0	0	0	0	0	0	0	0	0	0	0
10^{-6}	0	0	0	0	0	0	0	0	0	0	0	0	0	0	0	0
10^{-7}	0	0	0	0	0	0	0	0	0	0	0	0	0	0	0	0
10^{-8}	0	0	0	0	0	0	0	0	0	0	0	0	0	0	0	0

Note: Key: "+" = Virus recovery; "0" = No virus recovery and/or no cytotoxicity observed; "T" = Toxicity observed.

Supplementary Table S17. Test results for oral formulation D and nasal formulation E at a dilution of 1:32 against SARS-CoV-2.

Test Product	Concentration	Virus titer (TCID$_{50}$)	Log$_{10}$ reduction	% Reduction
Oral formulation D	1:32	5.485x10^{1}	>4.000	>99.99%
Nasal formulation E	1:32	4.39x10^{1}	>4.000	>99.99%
Tested Virus	SARS-CoV-2			
Virus Control	1.2x10^{6} TCID$_{50}$			
Virus recovery	6.10x10^{5}			
Cytotoxicity Control	No cytotoxicity observed			
Sterility Control	No contamination observed			

Supplementary Table S18. Virus recovery data for SARS-CoV-2.

Dilution	Virus control set 1				Virus control set 2				Virus control set 3				Virus control set 4			
10^{-1}	+	+	+	+	+	+	+	+	+	+	+	+	+	+	+	+
10^{-2}	+	+	+	+	+	+	+	+	+	+	+	+	+	+	+	+
10^{-3}	+	+	+	+	+	+	+	+	+	+	+	+	+	+	+	+
10^{-4}	+	+	+	+	+	+	+	0	+	+	+	+	+	+	+	0
10^{-5}	+	0	0	0	+	+	0	0	+	0	0	0	+	0	0	0
10^{-6}		0	0	0	0	0	0	0	0	0	0	0	0	0	0	0
10^{-7}	0	0	0	0	0	0	0	0	0	0	0	0	0	0	0	0
10^{-8}	0	0	0	0	0	0	0	0	0	0	0	0	0	0	0	0
Log	5.62x10^{5}				1.00x10^{6}				5.62x10^{5}				3.16x10^{5}			
Average	3.43x10^{5}															

Supplementary Table S19. Virucidal activity of oral formulation D at a dilution of 1:32 against SARS-CoV-2.

Dilution	Virus-test suspension set 1				Virus-test suspension set 2				Virus-test suspension set 3				Virus-test suspension set 4			
10^{-0}	+	+	+	+	+	+	+	+	+	+	+	0	+	+	+	+
10^{-1}	+	+	0	0	0	0	0	0	0	0	0	0	+	0	0	0
10^{-2}	0	0	0	0	0	0	0	0	0	0	0	0	0	0	0	0
10^{-3}	0	0	0	0	0	0	0	0	0	0	0	0	0	0	0	0
10^{-4}	0	0	0	0	0	0	0	0	0	0	0	0	0	0	0	0
10^{-5}	0	0	0	0	0	0	0	0	0	0	0	0	0	0	0	0
10^{-6}	0	0	0	0	0	0	0	0	0	0	0	0	0	0	0	0
10^{-7}	0	0	0	0	0	0	0	0	0	0	0	0	0	0	0	0
TCID$_{50}$	1.0x10^{2}				3.16x10^{1}				3.16x10^{1}				5.62x10^{1}			
Average	5.485x10^{2}															

Note: Key: "+" = Virus recovery; "0" = No virus recovery and/or no cytotoxicity observed; "T" = Toxicity observed.

Supplementary Table S20. Virucidal activity of nasal formulation E at a dilution of 1:32 against SARS-CoV-2.

Dilution	Virus-test suspension set 1				Virus-test suspension set 2				Virus-test suspension set 3				Virus-test suspension set 4			
10^{-0}	+	+	+	+	+	+	+	+	+	+	+	+	+	+	+	+
10^{-1}	0	0	0	0	0	0	0	0	+	0	0	0	+	0	0	0
10^{-2}	0	0	0	0	0	0	0	0	0	0	0	0	0	0	0	0
10^{-3}	0	0	0	0	0	0	0	0	0	0	0	0	0	0	0	0
10^{-4}	0	0	0	0	0	0	0	0	0	0	0	0	0	0	0	0
10^{-5}	0	0	0	0	0	0	0	0	0	0	0	0	0	0	0	0
10^{-6}	0	0	0	0	0	0	0	0	0	0	0	0	0	0	0	0
10^{-7}	0	0	0	0	0	0	0	0	0	0	0	0	0	0	0	0
$TCID_{50}$	$3.16x10^{1}$				$3.16x10^{1}$				$5.62x10^{1}$				$5.62x10^{1}$			
Average	$4.39x10^{1}$															

Note: Key: "+" = Virus recovery; "0" = No virus recovery and/or no cytotoxicity observed; "T" = Toxicity observed.
